# Supplementary material for: Mechanisms governing the pioneering and redistribution capabilities of the non-classical pioneer PU.1
Source: Nat Commun. 2020 Jan 21;11:402. doi: 10.1038/s41467-019-13960-2 (PMC6972792; doi:10.1038/s41467-019-13960-2)
Supplement: Supplementary file 7 — Source data [file 41467_2019_13960_MOESM7_ESM.zip › Source_Data/FigureS2/FigureS2B_MotifScanOutput/KmeansCluster3/homerResults/motif34.similar.html]

motif34

## Information for motif34

A
G
T
C
C
G
A
T
A
G
C
T
G
A
T
C
A
G
C
T
A
G
C
T
G
T
A
C
G
C
T
A
A
G
T
C
C
T
G
A
C
G
A
T
C
T
A
G
C
T
A
G
A
G
T
C
  
Reverse Opposite:  

T
C
A
G
G
A
T
C
A
G
T
C
G
C
T
A
A
G
C
T
C
T
A
G
C
G
A
T
A
C
T
G
C
T
G
A
C
T
G
A
C
T
A
G
C
T
G
A
G
C
T
A
C
T
A
G
  

|  |  |
| --- | --- |
| p-value: | 1e-27 |
| log p-value: | -6.393e+01 |
| Information Content per bp: | 1.755 |
| Number of Target Sequences with motif | 86.0 |
| Percentage of Target Sequences with motif | 1.80% |
| Number of Background Sequences with motif | 143.8 |
| Percentage of Background Sequences with motif | 0.34% |
| Average Position of motif in Targets | 93.1 +/- 42.5bp |
| Average Position of motif in Background | 106.3 +/- 76.1bp |
| Strand Bias (log2 ratio + to - strand density) | 0.5 |
| Multiplicity (# of sites on avg that occur together) | 1.00 |
| Motif File: | file (matrix) reverse opposite |

### Similar de novo motifs found

|  |  |  |  |  |  |  |  |
| --- | --- | --- | --- | --- | --- | --- | --- |
| Rank | Match Score | Redundant Motif | P-value | log P-value | % of Targets | % of Background | Motif file |
| 1 | 0.897 | G A T C C T G A A T G C A G T C G C T A A G C T A C T G C A G T C A T G C G T A C T G A C T A G G T C A | 1e-22 | -50.973215 | 1.49% | 0.29% | motif file (matrix) |
| 2 | 0.938 | A G T C G C T A A C G T C T A G C G A T C T A G C G T A C G T A C A T G C T G A G C T A C T A G | 1e-21 | -49.144758 | 1.55% | 0.33% | motif file (matrix) |
| 3 | 0.824 | A G T C T C A G A G T C A G T C C G T A A C G T A C T G A C G T A C T G C T G A T G C A | 1e-17 | -40.089191 | 1.49% | 0.38% | motif file (matrix) |
| 4 | 0.801 | A G T C C T A G A G T C A G T C C G T A A C G T A C T G A C G T A C T G C T G A | 1e-15 | -36.266785 | 3.00% | 1.31% | motif file (matrix) |
